# Investigation of the Charge Accumulation Based on Stiffness Variation of the Micro-Shell Resonator Gyroscope

**DOI:** 10.3390/mi14091755

**Published:** 2023-09-08

**Authors:** Mingze Gao, Jiangkun Sun, Sheng Yu, Jun Feng, Xingjing Ren, Yongmeng Zhang, Xuezhong Wu, Dingbang Xiao

**Affiliations:** College of Intelligence Science and Technology, National University of Defense Technology, Changsha 410073, China; gmz233@nudt.edu.cn (M.G.);

**Keywords:** micro-shell resonator gyroscope, charge accumulation, frequency split, stiffness axis

## Abstract

In capacitive microelectromechanical system (MEMS) devices, the application of dielectric materials causes long-term charging problems in the dielectric layers or substrates, which especially affect the repeatability and stability of high-performance devices. Due to the difficulties of observation and characterization of charge accumulation, an accurate characterization method is needed to study the effect of charge and propose suppression methods. In this paper, we analyze the influence of charge accumulation on the MSRG and propose a characterization method for charge accumulation based on stiffness variation. Experiments are carried out to characterize the charge accumulation in MSRG, and the effect of temperature on the process is also investigated. In the experiment, the charge accumulation is characterized accurately by the variation of the frequency split and stiffness axes. Furthermore, the acceleration of the charge accumulation is observed at high temperatures, as is the higher additional voltage from the charge accumulation.

## 1. Introduction

Capacitive structures for actuating and detecting are common in MEMS devices. Because of the existence of dielectric layers and substrates, the transfer of charge occurs between the metal electrodes and the dielectric. Under a stable electric field, these charges will eventually be trapped by the dielectric, causing charge accumulation in the dielectric. This phenomenon, also known as “dielectric charging”, has been observed in numerous devices and can have complex and long-term effects on MEMS devices.

The effect is first studied in a silicon cantilever actuator by detecting the deflection at both ends of the cantilever [1]. Similar effects were also found in several capacitive MEMS devices. In different materials, such as SiN_x_, SiO_2_, quartz, or some composite dielectric layers, the charge accumulation processes are different. Some materials and structures can accelerate the accumulation of charge, which provides ideas for the future solution of this problem [2]. The effects of temperature and humidity on charge accumulation in MEMS devices have also been observed [3,4]. In MEMS devices, it is hard to directly measure the charges in the dielectric layers. Therefore, a characterization method for the charge accumulation in the MEMS devices is required. In radio frequency (RF) MEMS switches, the charge accumulation is characterized by the C-V curve drift of the switch, and the decrease in cycle life caused by charge accumulation is observed [5,6,7]. In other MEMS devices, the long-term drift of output data was observed. In MEMS resonators, charge accumulation is often observed based on the drift of the resonant frequency [8,9,10]. For micromirrors, the long-term angular drift of the micromirror is used as a characterization of charge accumulation [11,12,13]. Up until now, charge accumulation has been difficult to accurately characterize, especially in devices with complex structures.

The MSRG is a capacitive MEMS inertial sensor known for its high stability, small size, and reliability. To meet the increasing demands for high-performance MEMS gyroscopes, it is essential to understand the error mechanisms and enhance the performance of the MSRG. By applying high bias voltages, the MSRG obtains a high signal-to-noise ratio and improves tunability. However, as the gyroscope’s accuracy improves, the application of high voltages also leads to significant charge accumulation, which limits the performance improvement of the gyroscope. To solve the current problem of charge accumulation and obtain high-performance gyroscopes, an accurate characterization of charge accumulation in MSRGs needs to be proposed.

In this paper, the stiffness variation is used to characterize the charge accumulation in the MSRG, which includes the frequency split and the angle of the stiffness axis. The relationship between stiffness and the additional voltage of charge accumulation is discussed, and the theoretical model is built. Experimental data show agreement with the model, indicating the presence of an additional voltage in the gyroscope. Moreover, the exponential law of voltage variation is demonstrated by the fitted voltage data. The effect of temperature changes on charge accumulation is also noted in the experiments. 

## 2. The Charge Accumulation in the MSRG

### 2.1. The Structure of MSRG

**Figure 1:**
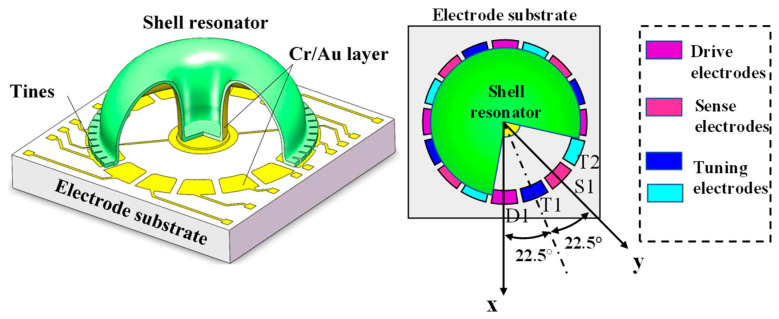
The MSRG structure and substrate electrodes.

The MSRG is a type of MEMS resonator with a micro-shell resonance structure, with a height and diameter of 4 mm and 12 mm, respectively. The resonance structure of the MSRG is composed of two parts bonded together with adhesive: a planar electrode substrate and a micro-shell resonator. Both components are made of fused silica to ensure low thermo-elastic damping and stable resonance performance. The micro-shell resonator made of fused silica operates at a high resonance frequency of 13 kHz, which results in a high Q factor and a ring-down time of 6 s. For more consistent performance, the resonator and substrate are packaged in a vacuum-filled metal shell. To drive the motion of the micro-shell resonator and measure its movement, circular Cr/Au electrodes are coated on the top surface of the substrate and also at the bottom of the micro-shell resonator [14]. The structure of MSRG is shown in Figure 1.

As the figure shows, the 16 electrodes on the substrate can be categorized into drive, sense, and tuning electrodes. The tuning electrodes T1 and T2 are positioned between the drive and sense electrodes, with each electrode separated by an angle of 22.5 degrees. Different voltages are applied to electrodes according to their functions. The applied voltages typically range from tens to several volts.

### 2.2. Charge Accumulation in MSRG

To explain the process of charge accumulation, several charge transportation theories have been proposed, such as the Poole–Frenkel effect, Schottky emission, and Fowler–Nordheim tunneling (FNT) theory [15]. In these theories, the current densities during charge transfer are related to the bias voltages and temperature. Generally, the accumulation process is described as an exponential model. For an idealized dielectric, the accumulated charge can be described by the model with a single time coefficient:

(1) $$\begin{array}{l} Q_{ch\arg e}=Q_{\max}(1-\exp(-t/\tau_{c}))\\ Q_{disch\arg e}=Q_{\max}(1-\exp(-t/\tau_{d})) \end{array}$$

where *Q*_max_ is the maximum charge, *τ_c_* and *τ_d_* are the time coefficients of the charging and discharging conditions, respectively. However, the actual charging process may contain multiple accumulation factors. In this case, exponential models with multiple time coefficients or stretching exponential models containing stretching coefficient *β* are utilized for description. The multi-exponential models and stretching exponential models are proposed as Equations (2) and (3), where *Q_i_* is the maximum charge, *τ_ci_* and *τ_di_* are the charging and discharging time coefficients for each part.

(2) $$\begin{array}{c} Q_{ch\arg e}=\sum_{i=1}^{n}Q_{i}(1-\exp(-t/\tau_{ci}))\\ Q_{disch\arg e}=\sum_{i=1}^{n}Q_{i}(1-\exp(-t/\tau_{di})) \end{array},$$

(3) $$\begin{array}{l} Q_{ch\arg e}=Q_{\max}(1-\exp{\left(-t/\tau_{c}\right)}^{\beta })\\ Q_{disch\arg e}=Q_{\max}(1-\exp{\left(-t/\tau_{d}\right)}^{\beta }) \end{array}.$$

**Figure 2:**
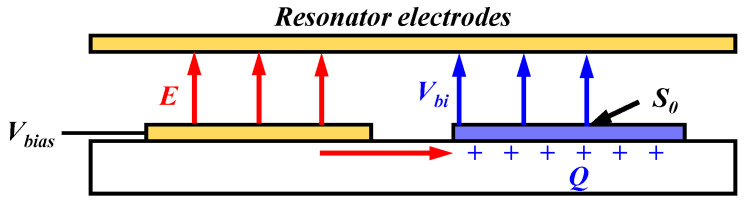
The charge accumulation in the substrate dielectric of the MSRG.

The structure of MSRP electrodes is shown in Figure 2. When the MSRG is working, the electric field *E* results in the movement and accumulation of the charges. The effect of the accumulated charges *Q* can be equal to an additional bias voltage *V_bi_*, which will change the stiffness of the resonator due to the electrostatic negative stiffness effect.

The effect of *V_bi_* on the stiffness of the resonator is:

(4) $$\Delta k=-\frac{S\varepsilon }{d^{2}}V_{bi}^{2}$$

where *d* represents the electrode gap of the MSRG, *ε* is the vacuum dielectric constant, and *S* represents the area of charge accumulation. When a floating electrode *S*_0_ exists, the charges mainly accumulate in the substrate under the electrode; therefore, *S* can be equated to the area of the floating electrode.

## 3. The Stiffness Variation of MSRG under the Charge Accumulation

### 3.1. The Stiffness Mismatch of MSRG

**Figure 3:**
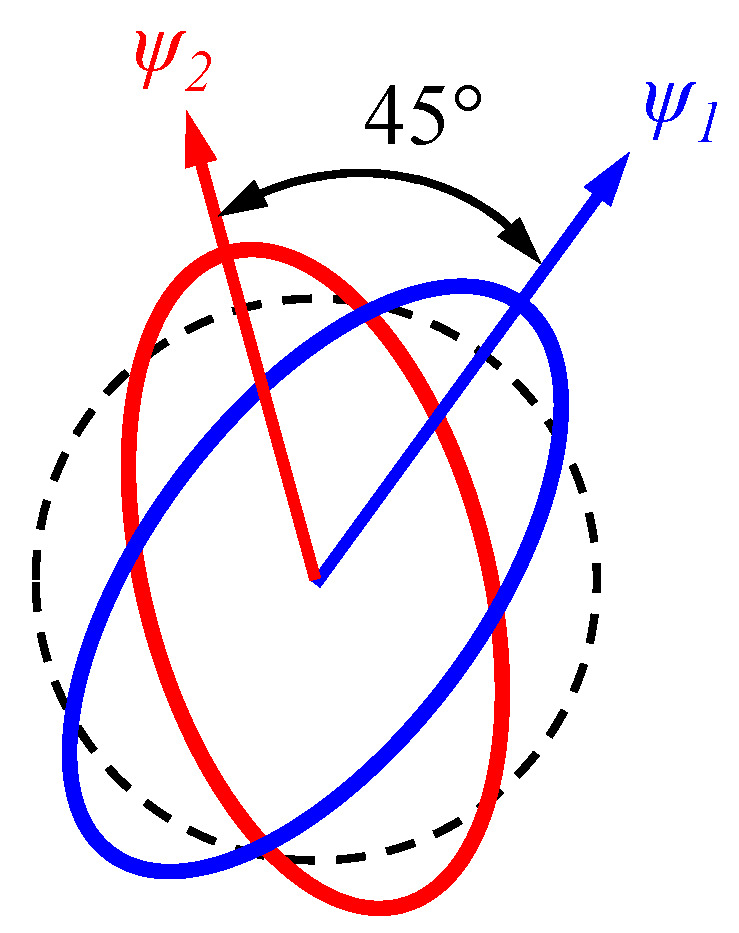
The two vibration patterns of the MSRGs n = 2 mode.

The MSRG operates in the n = 2 vibration mode, specifically known as the "wineglass mode." In this mode, the shell resonator vibrates in an elliptical pattern consisting of two modes with identical frequencies, assuming ideal conditions. The included angle between the two vibration patterns is 45°. Figure 3 illustrates the relationship between these two vibration patterns, with the azimuth angle of the high-frequency pattern denoted as *Ψ*_1_. Consequently, the azimuth angle of the low-frequency vibration pattern, *Ψ*_2_, can be expressed as:

(5) $$\psi_{2}=\psi_{1}+45{}^{\circ}$$

The errors in the fabrication and assembly of the gyroscope contribute to the inherent stiffness mismatches of the MSRG. At this point, the resonant frequencies of the two vibration modes are no longer equal but split into the maximum and minimum frequencies *ω*_1_ and *ω*_2_. The difference between the two frequencies is defined as the “frequency split.”

(6) $$\Delta \omega =\omega_{1}-\omega_{2}$$

### 3.2. The Stiffness Variation under Charge Accumulation

Based on the model of the mass and stiffness variations in the resonant structure [16], the stiffness variation of MSRG is discussed and the theoretical model is built.

**Figure 4:**
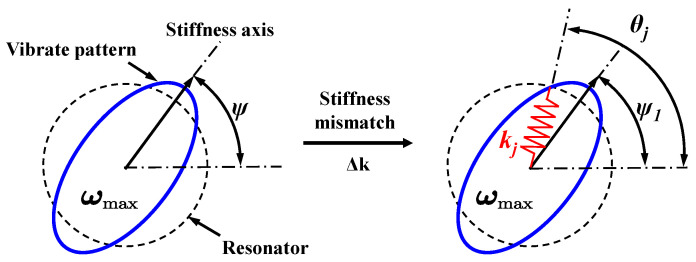
Frequency split model of the MSRG resonator.

The ideal resonator with the vibrating pattern is represented in Figure 4. The primary axis of the vibration pattern aligns with the stiffness axis, which has an angle *ψ*. In practical resonators, there are mismatches in mass and stiffness, resulting in an initial frequency split known as Δ*ω*_0_, and a stiffness angle denoted as *ψ*_0_. The additional bias voltage resulting from charge accumulation introduces variations in the stiffness of the resonator. These variations can be modeled as the addition of n radial springs, each with stiffness *k_j_* and azimuths *φ_j_.* The resulting frequency split Δ*ω*_1_ and stiffness angle *ψ*_1_ can be expressed as Equation (7):

(7) $$\Delta \omega_{1}e^{i4\psi_{1}}=\Delta \omega_{0}e^{i4\psi_{0}}+\lambda_{k}\sum_{j=1}^{N_{k}}k_{j}e^{i4\phi_{j}}$$

where *λ_k_* is a coefficient related to the mechanical properties of the resonator.

According to Equations (4) and (7), the variation in the stiffness of MSRG when charge accumulation occurs is presented as follows:

(8) $$\begin{array}{c} \Delta \cos4\psi_{1}=\Delta_{0}\cos4\psi_{0}+\alpha \cos4\phi_{j}\cdot V_{bi}^{2}\\ \Delta \sin4\psi_{1}=\Delta_{0}\sin4\psi_{0}+\alpha \sin4\phi_{j}\cdot V_{bi}^{2} \end{array},$$

where the *α* can be present as the following equation:

(9) $$\alpha =-\lambda_{k}\frac{S\varepsilon }{d^{2}}$$

Since the coefficient *α* is only related to the mechanical property of the MSRG, it remains constant during a charge accumulation process. Thus, if an accurate variation of stiffness can be obtained, the theoretical model can be used for the characterization of the additional voltage.

## 4. The Measurement of Stiffness Mismatch Based on Self-Precession

**Figure 5:**
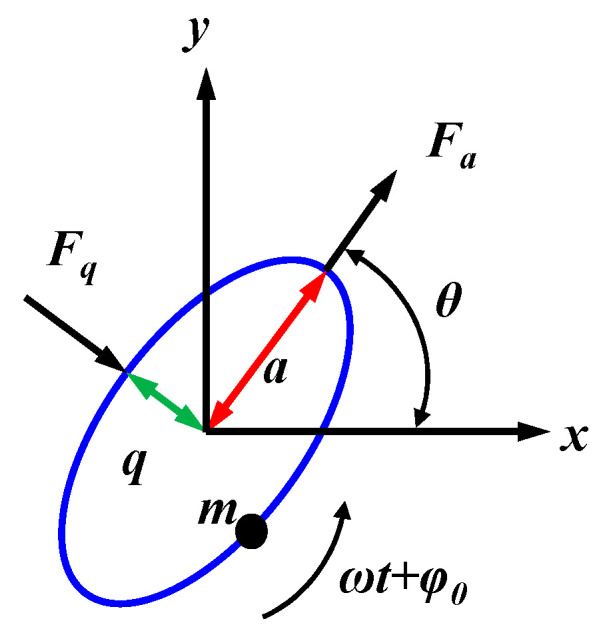
The elliptic solution of the motion of the gyroscope.

By applying the method of self-precession, the stiffness variation of MSRG can be accurately characterized. Self-precession is an operating condition of gyroscopes under rate-integrating control. In this condition, the driving force *f_qs_* is applied to the MSRG, making the vibrating pattern progress at a stable rate. According to Lynch’s method of average [17], the whole-angle mode can be expressed as an elliptical orbit shown in Figure 5 with major axis *a*, minor axis *q*, pattern angle, *θ* and vibrating phase *ωt + φ*. The vibrations can also be expressed as

(10) $$\begin{array}{l} x=a\cos\theta \cos(\omega t+\phi_{0})-q\sin\theta \sin(\omega t+\phi_{0})\\ y=a\sin\theta \cos(\omega t+\phi_{0})-q\cos\theta \sin(\omega t+\phi_{0}) \end{array}$$

The vibrating pattern of the resonator will pulse under the angular rate Ω. During precession, the expression of the main control variables of MSRG is:

(11) $$\begin{array}{l} E=a^{2}+q^{2}=c_{x}^{2}+s_{x}^{2}+c_{y}^{2}+s_{y}^{2}\\ Q=2aq=2(c_{x}s_{y}-c_{y}s_{x})\\ R=(a^{2}-q^{2})\cos2\theta =c_{x}^{2}+s_{x}^{2}-c_{y}^{2}-s_{y}^{2}\\ L=(a^{2}-q^{2})\sin2\theta =2(c_{x}c_{y}-s_{y}s_{x})\\ \theta =\frac{1}{2}\arctan\frac{S}{R} \end{array}$$

where *E* represents the energy of the system and *Q* represents the orthogonal quantity. *R* and *S* are variables representing the cosine and sine, respectively, which are used to calculate the angle of the vibration mode *θ* in the resonant structure. *L* represents the phase error of the input and output on the MSRG. The *s_x_*, *s_y_*, *c_x_*, and *c_y_* are the in-phase and quadrature components in the *x*-axis and *y*-axis directions of the detection signal, respectively.

The *F_a_* and *F_q_* represent the effective forces applied at the major and minor axes of the ellipse. The force can be decomposed into the in-phase and quadrature components as follows:

(12) $$\begin{array}{l} F_{a}=f_{ac}\cos(\omega t+\phi )+f_{as}(\omega t+\phi )\\ F_{q}=f_{qc}\cos(\omega t+\phi )+f_{qs}(\omega t+\phi ) \end{array}$$

The MSRG under rate-integrating control has four main control loops: the energy control loop, the quadrature control loop, the angle control loop, and the phase control loop.

The angular control loop of the gyroscope is shown in Equation (3). The Ω is the input angular rate, *ω* is the resonance frequency, *κ* is the coefficient between $\dot{\theta }$ and Ω. The second and third terms of the equation represent the damping and stiffness drift, respectively.

(13) $$\dot{\theta }=-\kappa \Omega +\frac{1}{2}\Delta (\frac{1}{\tau })\sin2(\theta -\theta_{\tau })+\frac{1}{2}\Delta \omega \cos2(\theta -\theta_{\omega })\frac{Q}{E}-\frac{f_{qs}}{2\omega \sqrt{E}}$$

where *Q* is the quadrature error and *E* is the energy of the MSRG. *θ_τ_*, *θ_ω_* are respectively, the damping angle and stiffness angle. In the following test, a constant size of *f_qs_* can be applied to the gyroscope to obtain a stable vibration mode that progresses at a constant rate. That is, without the input of angular rate, the virtual mode self-precession is realized through the control force *f_qs_*, and the precession rate Ω*_s_* is:

(14) $$\Omega_{s}=\frac{f_{qs}}{2\omega \sqrt{E}}$$

The self-precession method is widely used in the MSRGs performance testing and parameter tuning. For the resonator, when the vibrate pattern precesses at a constant rate with a period of *π*, the resonant frequency of the gyroscope at different modal angle *θ* can be presented as Equation (15):

(15) $$\omega =\omega_{0}-\frac{1}{2}\Delta \omega \cos2(\theta -\theta_{\omega })$$

**Figure 6:**
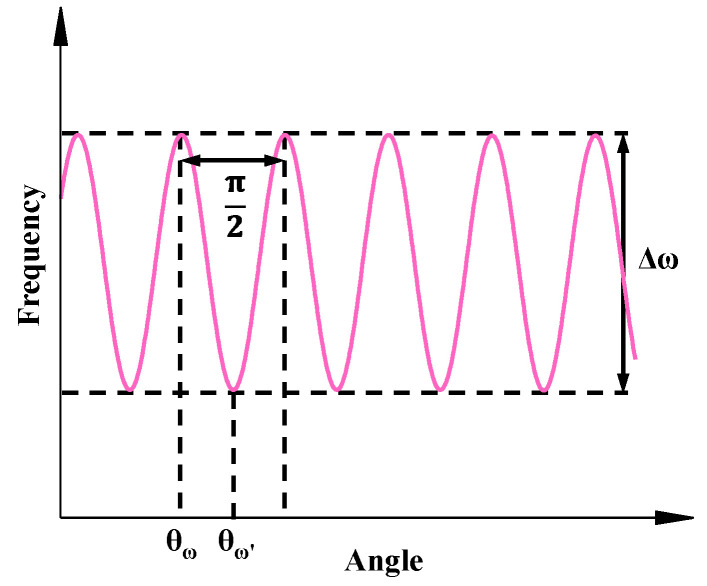
Identification results of frequency splitting and stiffness axis.

The frequencies of the mode angles with a period of π/2 are shown in Figure 6.

The difference between the highest and lowest points of the frequency-angle curve is the frequency split Δ*ω*. At the same time, the angles corresponding to the highest point and the lowest point are the stiffness axis angles of two vibration modes, which are the semimajor axis azimuths of the vibrating patterns.

In the experiments, by periodically recording the frequency and angle signals of the MSRGs output, an accurate frequency split and stiffness axis angle can be obtained and used for the characterization of charge accumulation.

## 5. Characterization of Charge Accumulation Based on the Stiffness Variation

### 5.1. The Calibration of the Theoretical Model

**Figure 7:**
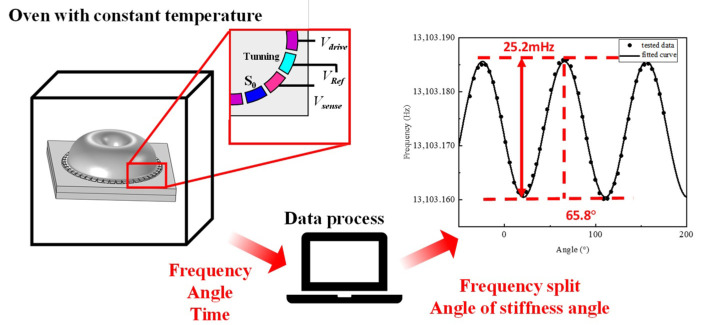
The experiment set of model calibration experiments.

After characterizing the stiffness variations, the characterization experiments with additional voltage can be accomplished. Before the characterization, the coefficients should be calibrated in the following experiments. The details of the characterizing experiment is shown in Figure 7.

During the test, the gyro is placed in an oven to keep the temperature constant and avoid the effect of temperature drift during gyro operation. Meanwhile, in order to avoid interference from the reference voltage applied at the resonator electrode, the same magnitude of Vref is applied at the other trimming electrodes. After the test starts, the gyro is first made to operate for a certain period of time at a constant temperature of 30 °C, and then the bias voltage Vtest is applied at the S0 electrode to simulate the effect of the additional bias voltage generated by the charge accumulation on the gyro. The frequency and angle of the gyro output were collected and fitted to the corresponding frequency split and angle of the stiffness axis during the experiment, with the voltage varying from 0 V to 17 V.

**Figure 8:**
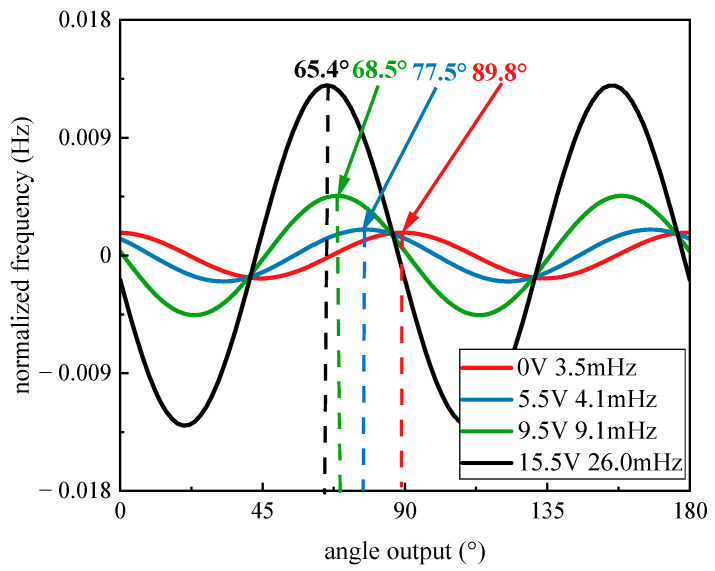
The frequency with angle under different bias voltages.

When the temperature is set to 30 °C, the tested data under several different voltages is shown in Figure 8. Finally, the coefficient is calculated with the tested data. With the increase in test voltage, the drift of the frequency split and angle of the stiffness axis occurs. The frequency split varied from 3.5 mHz to 26 mHz, while the angle of the stiffness axis drifted by almost 30 degrees.

**Figure 9:**
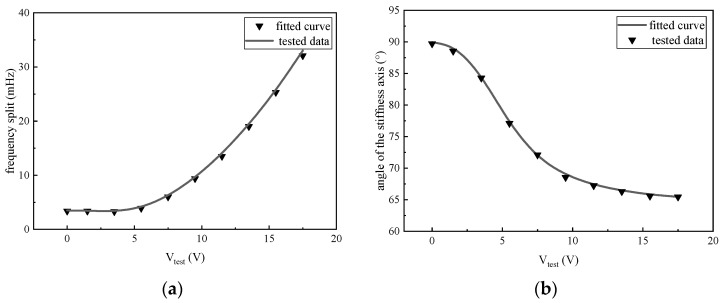
Comparison of frequency difference (**a**) and stiffness axis deflection angle (**b**) between the curve obtained from model fitting and the tested data.

The comparison between the data fitted from the model and the experiment is shown in Figure 9. It can be seen that both the frequency split and the stiffness axis angle from the experiment can be properly matched with the model. As the figures show, different additional voltages under the floating electrode *S*_0_ affect the frequency split and angle of the stiffness axis. For voltages higher than 5 V, an influence on the frequency split is observable. When the voltage is lower than 5 V, the main effect is the angle drift of the stiffness axis, while the change of the frequency split is hard to observe. However, considering the experimental error and the random drift from the gyroscope, both the angle drift and the frequency split may be hard to observe for voltages lower than 2 V since only less than 2 degrees are changed.

**Figure 10:**
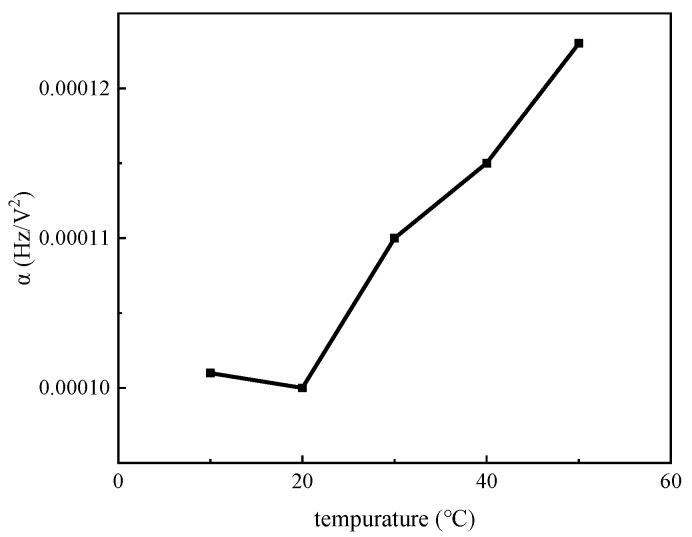
The charge accumulation coefficient that changes with temperature.

For temperatures above 20 °C, the value of α increases with the temperature. The relationship between α and temperature is illustrated in Figure 10. By calibrating this parameter at different temperatures, it is possible to accurately characterize charge accumulation at different temperatures.

### 5.2. The Characterization of Charge Accumulation with Stiffness Variation 

After the calibration of the model, the characterization of the additional voltage from charge accumulation was successfully achieved at a constant temperature of 30 °C.

The calibration experiment is completed as follows: Firstly, MSRG is placed in the oven for a long enough time, and the test electrode *S*_0_ is kept grounded. Secondly, the electrode *S*_0_ is kept floating, and the charge accumulation process is measured until the stiffness axis angle and frequency split of the gyroscope become stable, at which time the charge accumulation under the floating electrode is considered to reach a stable condition. Finally, the additional bias *V_bi_* with the recorded data are calculated.

**Figure 11:**
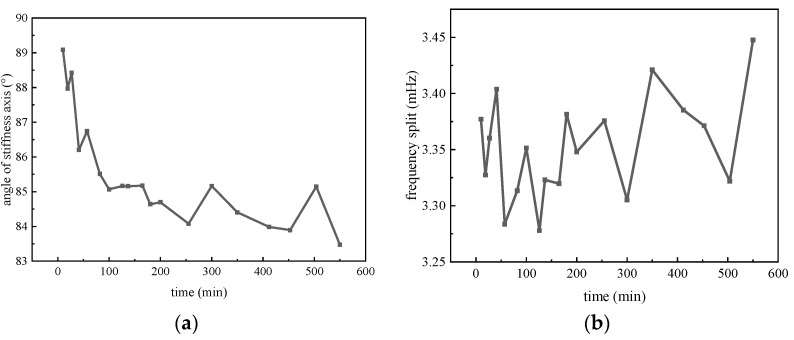
Measured frequency split and high frequency angle of MSRG. (**a**) Variation of the angle of stiffness axis with time; (**b**) Variation of the angle of stiffness axis with time.

The tested angle of the stiffness axis and the frequency split are shown in Figure 11. Throughout the 600 min testing, a long-term drift in the stiffness angle is observed. However, there is no significant variation in the frequency split. The drift speed decreases with time, and the angle ultimately stabilizes at 84 degrees. As for the frequency split, it remains consistently stable at 3.35 mHz. The results indicate that the stiffness axis angle of the gyroscope is more susceptible to the influence of charge accumulation due to the small additional bias voltage generated.

**Figure 12:**
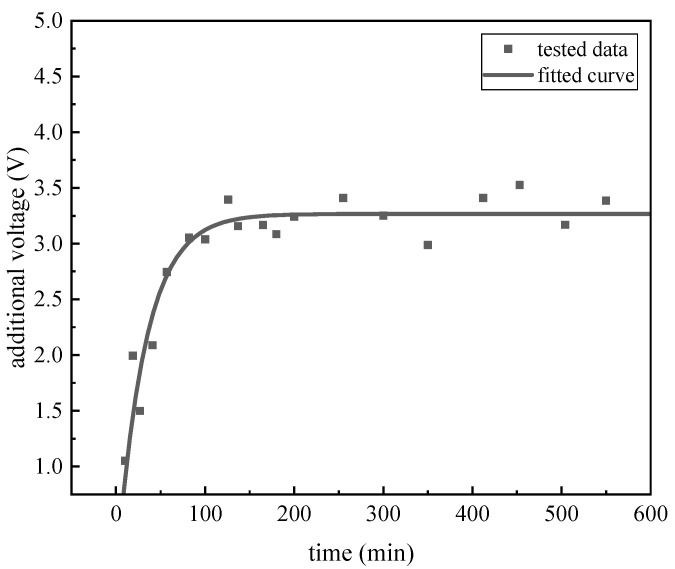
The charge accumulation under 30 °C.

The additional bias voltage of the charge accumulation is calculated and shown in Figure 12. The voltage is calculated every 10 min, and the exponential model is fitted to the measured points. The variation of the additional bias voltage is found to match the change law of a single exponential model. As the model described, the voltage is increasing with time, and it also conforms to the change from fast to slow, which is similar to the variation of stiffness angle. The voltage that is fitted by the exponential model is as follows:

(16) $$V_{bi}=3.267(1-\exp(-t/32.05))$$

At 200 min after the start of the experiment, the additional bias voltage caused by charge accumulation tends to be stable, which also means that the charge accumulation has been saturated. It can be seen that the charge accumulation in the floating electrode of MSRG is a long-term process, which will affect the repeatability of the gyroscope for hundreds of minutes at room temperature. In addition, the r-squared goodness of fit of the single exponential voltage model is 0.9076, which proves the validity of the charge accumulation characterization method, while the charge accumulation process of a single suspended electrode can be well described by the exponential model.

### 5.3. Characterization Experiments with Temperature Effects

**Figure 13:**
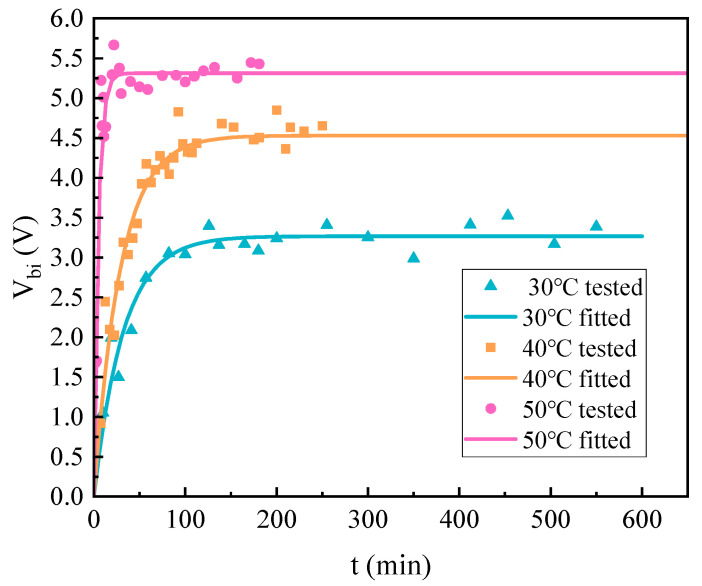
The charge accumulation process at different temperatures.

In order to accomplish a more accurate characterization of charge accumulation, the effects caused by temperature changes need to be clarified. The experimental procedure for characterizing charge accumulation was repeated by changing the temperature of the above experiment, and the charging processes under different temperatures were calculated. Figure 13 and Equation (17) show the experimental results of the additional voltages:

(17) $$\begin{array}{l} V_{40}=4.70(1-\exp(-t/14.24))\\ V_{50}=5.31(1-\exp(-t/4.84)) \end{array}$$

According to the experimental results, the process of charge accumulation shows a clear trend of change with temperature. As the temperature increases, the charge accumulates in a shorter time, and the final additional bias voltage increases. This result indicates that the rate and amount of charge accumulation are both temperature-dependent. Under high-temperature conditions, the time required for charge accumulation to stabilize is shorter, and more charges can be captured in the dielectric.

However, for conditions at 20 °C and 10 °C, no significant stiffness axis shift was observed during hundreds of minutes of testing, indicating that the influence of charge accumulation on stiffness can be ignored in low-temperature environments.

**Figure 14:**
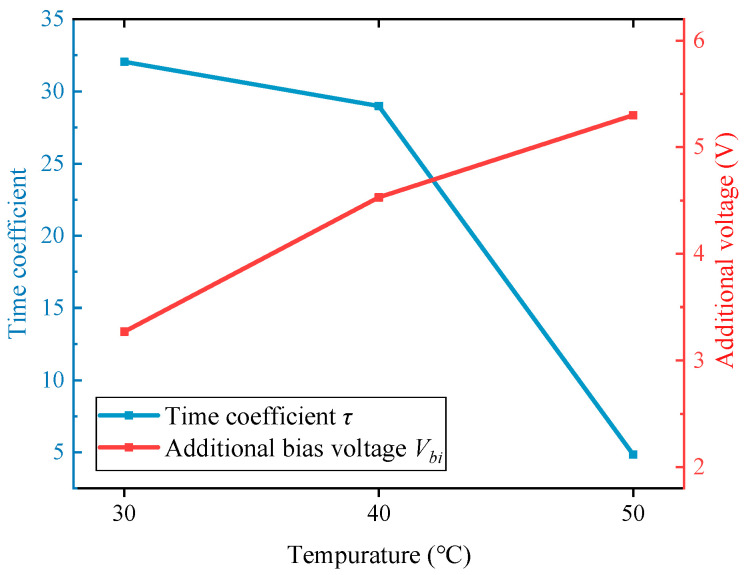
Coefficients of charge accumulation vary with temperature.

As Figure 14 shows, the coefficients in the exponential model of the additional voltage vary with the temperature. The amount of accumulated charge varies linearly, while the accumulation time constant tends to change in an exponential-like form. This suggests that the accumulated charge increases as the temperature rises; however, at the same time, the rate of accumulation accelerates rapidly. In addition, the reason no significant effect was observed at temperatures lower than 20 °C may be due to the measurement errors and random drifts of the gyroscope, which mask the small perturbations to the stiffness axis and frequency split.

## 6. Conclusions

The charge accumulation limits the improvement of MEMS devices’ performance. However, the lack of characterization methods for charge accumulation in MEMS devices, especially MEMS gyroscopes, limits the presentation of suppression methods. We analyze the charge accumulation in MSRGs using stiffness variation and propose a theoretical model for the effect of charge accumulation on stiffness in the MSRG. The additional bias voltage for charge accumulation can be characterized by stiffness variations inside the MSRG, such as frequency split variations and variations in the angle of the stiffness axis of the resonator. 

In the experiments, the accuracy of the theoretical model for characterizing charge accumulation is verified. An additional bias voltage of about 3 V is generated in the substrate dielectric of the MSRG at a constant temperature of 30 °C. The accumulation process lasts for more than 200 min and the variation of the additional bias voltage is observed to be consistent with an exponential model for a single factor. Similar accumulations are observed at 40 °C and 50 °C, and the temperature effect is tested. As the temperature increases, the time used for charge accumulation becomes dramatically shorter, while the final additional bias voltage increases linearly. At 50 °C, the charge accumulation process takes only 20 min to stabilize. The results show that the effect of charge accumulation cannot be neglected in MSRG, especially in the presence of floating electrodes. It is also important to note the effect of temperature changes on charge accumulation, which becomes fiercer at higher temperatures and has a longer effect at ordinary temperatures. 

Charge accumulation is a significant problem that limits the performance improvement of MEMS devices. Accurate characterization of the extra bias voltage in the dielectric can be achieved by our proposed method, which lays the foundation for subsequent research on charge accumulation suppression based on methods such as bipolar driving. In the follow-up, the model can be subsequently extended to characterize the discharge process; however, this may require a separate design of experiments.

## Data Availability

Not applicable.
